# A larger target leads to faster evolution

**DOI:** 10.7554/eLife.62689

**Published:** 2020-10-14

**Authors:** Bing Yang, Scott A Rifkin

**Affiliations:** Section of Ecology, Behavior and Evolution, Division of Biological Sciences, University of California, San DiegoSan DiegoUnited States

**Keywords:** mutational variance, vulva, *Caenorhabditis*, evolutionary rate, mutational target

## Abstract

The speed at which a cell fate decision in nematode worms evolves is due to the number of genes that control the decision, rather than to a high mutation rate.

**Related research article** Besnard F, Picao-Osorio J, Dubois C, Félix MA. 2020. A broad mutational target explains a fast rate of phenotypic evolution. *eLife* **9**:e54928. doi: 10.7554/eLife.54928

Evolution is a two-step process: first, variation is generated, for example through random mutations; and then events such as natural selection determine whether specific traits become more or less common in a population. Some traits evolve faster than others, and different explanations for this emphasize the relative importance of one or the other of the two steps. For example, a fast-evolving trait could be under sustained and intense selection pressure, so that when a favorable new variant arises, it quickly spreads throughout the population. Alternatively, mutations might affect certain traits more than others, leading to more variation in these traits. Since variation is the fuel of evolution, this could make the affected traits change faster.

At the molecular level, two mechanisms can bias which traits are more likely to be affected by mutations. On one hand, the genes that affect a particular trait could be located in genomic areas with high mutation rates, known as ‘mutational hotspots’ (Fondon and Garner, 2004; Xie et al., 2019); on the other, a phenotype could depend on a large number genes, increasing the ‘mutational target size’ (Boyle et al., 2017).

*Caenorhabditis elegans* and *C. briggsae* worms have two sexes – males, which are very rare, and hermaphrodites. In hermaphrodites, the cell divisions and fate decisions that occur during development are nearly identical for individuals within a species. However, a cell called P3.p, which is involved in the development of the vulva, sometimes divides and sometimes does not, even among worms with the same genome (Sulston and Horvitz, 1977). A decade ago Marie-Anne Félix and colleagues reported that the frequency of this binary decision evolves more quickly than the cell fate decisions made by other early vulval cells, and that this fast evolution was probably due more to mutational biases – either hotspots or large target size – than selection effects (Braendle et al., 2010). Now, in eLife, Fabrice Besnard, Joao Picao-Osorio, Clément Dubois and Félix – who are based at laboratories in Lyon and Paris – report that this mutational bias is caused by a large mutational target size (Besnard et al., 2020).

To show this, Besnard et al. used mutation accumulation lines of either *C. elegans* or the closely related *C. briggsae*. These lines started from the offspring of a single individual and were bred in parallel. All of the lines were inbred: in each line a single selfing hermaphrodite was the parent of the next generation. This meant that if the parent had a new mutation, there would be a good chance that its offspring would inherit it. In this environment, the effects of natural selection were minimized because the only requirement for a line to endure was that a single hermaphrodite from that line survived and reproduced. After many generations, each line accumulated its own constellation of new mutations. Since the lines all started out genetically identical and selection was minimal, any differences in traits between the lines were be due to the different mutations.

The mutation accumulation lines used in these experiments were evolved for 250 generations (Baer et al., 2005), after which Besnard et al. chose to look at five lines in which the P3.p cell fate changed the most. By combining genomic sequencing and gene modification through CRISPR technology, they identified one gene in each line that was responsible for the change and showed that: (i) none of the genes are in a mutational hotspot in the genome; and (ii) only one of these genes was known to have a role specifically in vulval development. This suggests that the mutational target for P3.p cell fate is much broader than previously thought (Figure 1).

**Figure 1.**
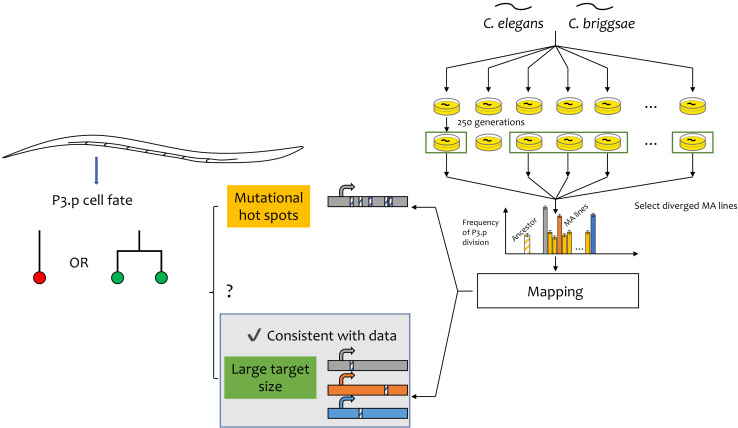
Fast evolution of a cell-fate decision in *C. elegans* and *C. briggsae*. The vulva in *Caenorhabditis* worms develops from six precursor cells. In some individuals, the most anterior of these cells (called P3.p) divides during development; in other worms it does not (left). The chance of it dividing differs between species. Cell division in the other five vulval precursor cells (VPCs; left) does not vary. This faster evolution of P3.p may be due due to mutational hotspots (if the genes that regulate this decision have a relatively high mutation rate) or to a large target size (if there are relatively more genes that affect this decision). Besnard et al. used a mutation accumulation (MA) approach in two species – *C. elegans* and *C. briggsae* – to distinguish between the two explanations (right). They examined the MA lines in which the decision frequency differed the most from the ancestor (shown by the vertical grey, orange and blue bars in the graph) and identified the genes responsible (shown by the horizontal grey, orange and blue bars at the bottom of the figure). They found that none of these genes appear to be in a mutational hotspot and that they have a range of different biological roles. In this case, fast evolution is due to a large mutational target size.

This broad mutational target is consistent with many genome-wide association studies and genetic mapping studies (Manolio et al., 2009; Shi et al., 2016). These experiments suggest that trait evolution tends not to be caused by one or two mutations with very large effects: rather, tens (or even hundreds) of genes carrying mutations with small effects seem to be responsible. These previous studies also suggest, rather unexpectedly, that a large fraction of the genes that underlie trait variation often have very little or no previously known functional relationships to the traits, and this is also the case in the work of Besnard et al. Therefore, there is a discrepancy between genetic pathways that have been described in the last century as being responsible for specific traits and the genes whose variation apparently fuels evolution.

The use of *Caenorhabditis* species in this work reinforces the power of this model system in research on developmental and evolutionary biology. Because so much is known about worm cell fates, Besnard et al. were able to go beyond identifying genes and pose testable hypotheses for why this particular cell was more variable than other vulval precursors. For example, they point out that the P3.p cell is at the far end of a gradient of cell fate-inducing molecules that are secreted from the tail end of the animal. The Félix lab previously showed that P3.p cell division is much more sensitive to variations in the dose of these molecules than cells closer to the source of the gradient (Pénigault and Félix, 2011). This suggests that the decision by P3.p to divide or not operates close to a concentration threshold for these molecules. This in turn means that mutations with small effects on the shape of the gradient, or on the responsiveness of P3.p to the molecules, could have large effects on the fate of the P3.p cell.

Developmental processes are responsible for generating the effects of many mutations. Therefore, to fully understand how mutations influence evolution, we must first understand the developmental context in which they occur.
